# Giving birth on the way to the clinic: undocumented migrant women’s perceptions and experiences of maternal healthcare accessibility along the Thailand–Myanmar border

**DOI:** 10.1186/s12978-023-01722-9

**Published:** 2023-12-06

**Authors:** Naomi Tschirhart, Wichuda Jiraporncharoen, Chaisiri Angkurawaranon, Ahmar Hashmi, Sophia Hla, Suphak Nosten, Rose McGready, Trygve Ottersen

**Affiliations:** 1 Interdisciplinary School of Health Sciences, Faculty of Health Sciences, University of Ottawa, Ottawa, Canada; 2 Department of Family Medicine, Chiang Mai University, Chiang Mai, Thailand; 3 Global Health and Chronic Conditions Research Group, Chiang Mai University, Chiang Mai, Thailand; 4 Institute for Implementation Science, University of Texas Health Sciences Center (UTHealth), Houston, TX USA; 5 Department of Health Promotion and Behavioral Sciences, School of Public Health, University of Texas Health Sciences Center (UTHealth) Houston, Houston, TX USA; 6 Mae Tao Clinic, Mae Sot, Tak Thailand; 7 Borderland Health Foundation, Mae Sot, Tak Thailand; 8 Centre for Tropical Medicine and Global Health, Nuffield Department of Medicine, University of Oxford, Oxford, UK; 9 Oslo Group on Global Health Policy, Department of Community Medicine and Global Health and Centre for Global Health, Institute of Health and Society, Faculty of Medicine, University of Oslo, Oslo, Norway; 10 Division for Health Services, Norwegian Institute of Public Health, Oslo, Norway; 11 Faculty of Medicine, Chiang Mai University, 110 Intawaroros Road, Si Phum, Muang, Chiang Mai, 50200 Thailand

**Keywords:** Maternal health services, Prenatal care, Birth, Healthcare, Pregnant, Migrant, Undocumented, Universal health coverage, Accessibility

## Abstract

**Background:**

Millions of women give birth annually without the support of a trained birth attendant. Generally and globally, countries provide maternal health services for their citizens but there is a coverage gap for undocumented migrant women who often can’t access the same care due to their legal status. The objective of this investigation is to explore undocumented migrants’ experiences and perceptions of maternal healthcare accessibility.

**Methods:**

We held focus groups discussions with 64 pregnant women at 3 migrant health clinics on the Thailand–Myanmar border and asked how they learned about the clinic, their health care options, travel and past experiences with birth services. In this context undocumented women could sign up for migrant health insurance at the clinic that would allow them to be referred for tertiary care at government hospitals if needed.

**Results:**

Women learned about care options through a network approach often relying on information from community members and trusted care providers. For many, choice of alternate care was limited by lack of antenatal care services close to their homes, limited knowledge of other services and inability to pay fees associated with hospital care. Women travelled up to 4 h to get to the clinic by foot, bicycle, tractor, motorcycle or car, sometimes using multiple modes of transport. Journeys from the Myanmar side of the border were sometimes complicated by nighttime border crossing closures, limited transport and heavy rain.

**Conclusions:**

Undocumented migrant women in our study experienced a type of conditional or variable accessibility where time of day, transport and weather needed to align with the onset of labour to ensure that they could get to the migrant clinic on time to give birth. We anticipate that undocumented migrants in other countries may also experience conditional accessibility to birth care, especially where travel is necessary due to limited local services. Care providers may improve opportunities for undocumented pregnant women to access maternal care by disseminating information on available services through informal networks and addressing travel barriers through mobile services and other travel supports.

*Trial registration* The research project was approved by Research Ethics Committee at the Faculty of Medicine, Chiang Mai University (FAM-2560-05204), and the Department of Community Medicine and Global Health at the University of Oslo—Norwegian Centre for Research Data (58542).

## Plain Language Summary

Undocumented pregnant migrants have difficulties and limitations in accessing maternal health care services. Although the governments have tried to provide maternal health care services to all, there is still a gap in coverage among this population. This study explores how undocumented pregnant migrants perceive their ability to access maternal health care and share their experiences when utilizing it. We used focus groups to interview 64 pregnant women at three migrant health clinics on the Thailand–Myanmar border. We asked how they learned about the clinic, their health care options, travel, and past experiences with birth services. The results showed that they usually knew about care options from community members and trusted care providers. The limitations for the choice of alternate care were due to a lack of services close to their homes, limited knowledge of other services, inability to pay hospital fees, and difficulty traveling from their residence to the clinic. Therefore, we anticipate that undocumented migrants in other countries may also experience difficulties in accessibility to birth care, especially where travel is necessary due to limited local services. Care providers may improve opportunities for these migrants to access maternal care by disseminating information on available services through informal networks and addressing travel barriers through mobile services and other travel supports.

## Background

Undocumented migrants often fall outside the scope of national health insurance programs and have precarious entitlements and accessibility to healthcare. With over three times more estimated migrants (people not living in their country of birth) than in 1970; and 3.6% of the global population being migrants; migrant access to health care is a key component of achieving the Sustainable Development goals [1]. For pregnant migrant women who do not have the appropriate documentation, accessing healthcare can be challenging. Quality antenatal and birth services are internationally recognized as important to ensure positive outcomes for mother and child [2]. However, in many countries entitlement to care is often limited to citizens and migrants with valid visas, leaving undocumented pregnant women without guaranteed access to healthcare.

Accessibility, defined by Levesque et al. as the “opportunity to reach and obtain appropriate health care services in situations of perceived need for care”, occurs at the intersection of services provided by the health systems and the population’s ability to use them [3]. To date, few studies have reported on the perceptions or experiences of undocumented pregnant migrants who are accessing healthcare [4]. This is partly due to the documentation status of migrants not being specified in publications. Understanding how women who are undocumented learn about services, select care and utilize services is essential to help inform the development of responsive interventions to improve care for migrant populations.

Thailand is internationally well known for health insurance programs that include migrants [5]. The country offers migrants health insurance coverage through the Social Security Scheme (SSS) for workers in the formal economy and through the Migrant Health Insurance Scheme (MHIS) for those ineligibles for SSS. A policy change in 2014 made it necessary for migrants to obtain a work permit prior to enrolling in MHIS, thereby making it inaccessible to undocumented migrants [6]. In the Thailand–Myanmar border region, to meet the needs of undocumented migrants an NGO developed a health insurance scheme known as the The Migrant Fufundfundnd (M-FUND) which became operational in mid-2017 in Tak Province [7]. Nevertheless, the reach of such initiatives is limited as undocumented migrants have challenges accessing health services or have minimal financial resources, as daily wage workers, to meet the required premiums.

Migrants from Myanmar make up one of the largest economic migrant populations in Thailand at an estimated 2.1 million in 2019 [8]. Migrants began coming from Myanmar to Thailand in the early 1990s, where lax labor laws and limited enforcement of migrant registration and documentation allowed for large populations to grow in the border region [9–11]. In 2015 Tak, Thailand’s westernmost province bordering Myanmar, became one of the provinces nominated as a special economic zone (SEZ), a Thai Government initiative to promote investment through tax incentives, a regular supply of foreign workers, and infrastructure development, in areas with access to natural resources and cross-border trade routes. Inadequate legal protections or enforcement result in violations and abuse of economic, social and cultural rights in these zones [12]. Studies estimate that half of all migrants are registered, so the official counts represent a large underestimate of the size of the population [13]. An estimated 40–45% of migrants in Thailand are women [8], so a need for antenatal and birthing services are expected.

Myanmar’s maternal and neonatal mortality rates are amongst the highest in South-east Asia. Myanmar is a nation where there are barriers preventing access to health care including antenatal and birth services [14]. Most of the population resides in rural areas and are cared for largely by community health workers and auxiliary midwives who have minimal resources due to chronic underfunding of the health system [15]. Significant catastrophic expenditure has been reported following hospitalization for antenatal and delivery care.

In this article we start to address the gap in understanding of how undocumented migrant women learn about services, reach care and utilize services by exploring the experiences of women from Myanmar utilizing antenatal care at several migrant clinics in a border SEZ region of north-western Thailand. This qualitative investigation explores undocumented migrants’ experiences and perceptions of maternal healthcare accessibility [16].

## Methods

### Research design and epistemological stance

Our qualitative research project was designed with a constructivist approach, emphasizing the construction of meaning through the research process. We selected focus group discussions as the method of choice to facilitate conversations between participants. These have proven both culturally appropriate and successfully utilized by other studies in this setting.

### Data collection

To better understand migrant women’s experiences of maternal healthcare and perceptions on accessibility in May 2018 we conducted 10 focus group discussions (FGDs) with 64 pregnant migrant women.

### Setting

**Fig. 1:**
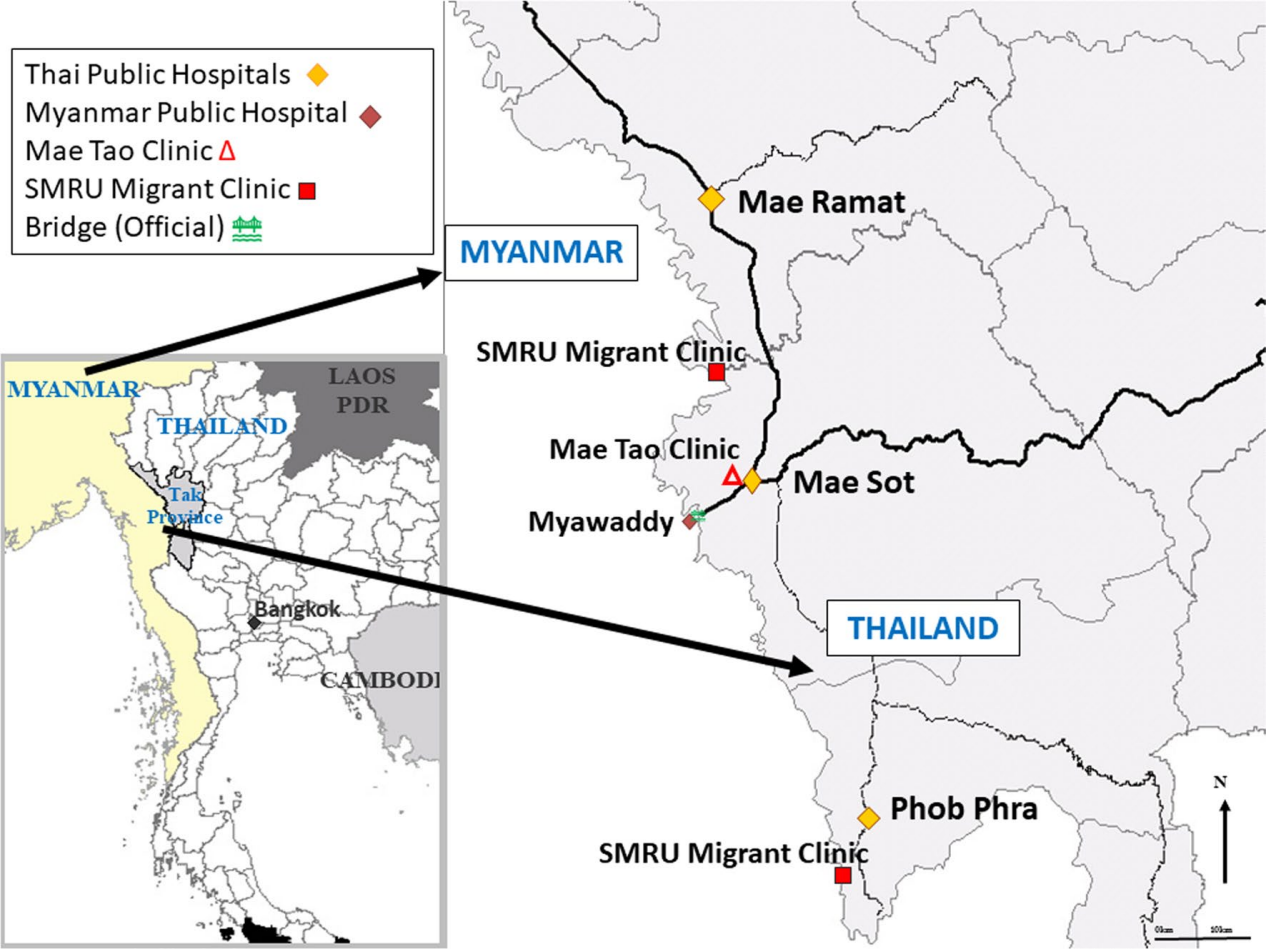
The location of healthcare facilities around Thai–Myanmar border

Three non-government clinics in rural areas along the Thailand–Myanmar border attended by undocumented migrants were included in the study. Clinics were run by non-profit and non-governmental entities providing care specifically to migrant populations and all have operated for more than 25 years. Mae Tao Clinic, near Mae Sot was opened in 1989, and Shoklo Malaria Research Unit established in 1986 in refugee camps, opened its first clinic for migrants in 1997. There are a larger number of public hospitals in Thailand on this section of the border than in Myanmar and the international border is demarcated by the Moei river (Fig. 1). Nighttime closures of the official border crossing bridge are a unique feature of this region which influences cross border movement and healthcare opportunities.

### Participant selection

We recruited women who were pregnant, identified as a non-Thai migrant, and spoke sufficient Burmese, Karen (Pwo or Sgaw) or Thai language to be able to be able to participate. Women who had an acute medical condition that would be disrupted or delayed by focus group participation were not eligible to participate. Despite Thailand’s efforts to provide affordable essential care for undocumented migrants through a voluntary prepayment scheme, only 33% of migrants were screened and enrolled into migrant health insurance in 2016 [17]. As a result, many undocumented migrants may not have had access to essential healthcare services. Undocumented status was not a requirement for inclusion in the study. We recruited women from clinics that primarily offer services to undocumented migrants and as a result many of the participants were undocumented. All of our participants had sought out clinic services for antenatal care and many were registered to give birth at the same clinic. In organizing discussion groups, we attempted to hold separate FGDs for first-time and experienced pregnant women. Participants were approached by health care staff while in the waiting area of the antenatal care department and explained briefly about the study and purpose and if they would like to learn more about the study or participate in the study. If a positive response was provided the private area where the FGD would take place was explained. Women were free to attend or not. Four to eight participants were expected per FGD. The sample size was planned based on previous experience with data saturation by the researchers with familiarity of the area.

Prior to beginning each focus group discussion, the interpreter (SN) read the consent form to participants who provided verbal consent. Women were informed they did not have to participate and could leave at any time, and were given the opportunity to leave. There were no drop outs. The facilitator (NT) documented that the consent had been received by signing the consent form. NT led the FGDs with interpretation and facilitation support from SN, an experienced community-based researcher fluent in English, Thai and Karen. AH, who conducted a prior maternal health study with a similar population, was present for most of the discussions. Focus groups lasted approximately 45 min. NT, SN and AH verbally debriefed after each group discussion.

A potential bias of this study is that we specifically enrolled undocumented migrants who had access to hospital care, thereby excluding those who did not have such access. This inclusion criteria may introduce selection bias if the characteristics of participants differ significantly from those of undocumented migrants who did not seek healthcare at the migrant clinics. Consequently, the study’s results cannot be extrapolated to the entire undocumented migrant population in the country.

### Focus group discussions

The FGD was based on a structured guide with questions and prompts. The questions in the guide were agreed upon by the research team following review of the literature on access to antenatal, birth and post-natal care and discussions with local qualitative researchers. Questions centered around their journey to care and decision making: why they chose to come to this specific clinic for ANC, how they found out about the clinic, their travel trajectory to the clinic, knowledge of other healthcare options and past experiences with birth services. During the focus group discussion we also conducted a discussion activity where we asked participants to select pictures that represent the most important factors when deciding where to give birth and we have reported on women’s birth decision making elsewhere [18]. Audio recording was used to collect the data with field notes made during and after the FGD. Transcripts were not returned to participants for comment or correction as reassembling the groups given antenatal schedules change with gestation and with delivery outcome.

### Data analysis

After the FGDs we had all of the audio files translated into English by a translator fluent in Karen and Burmese and English. Members of the research team met in person to discuss the coding frame which included codes for accessibility such as barriers and enablers, travel pathways, other options for care and health systems considerations. We uploaded the data into NVivo (version 12) software and conducted thematic analysis to code for predetermined themes of interest as well as emergent themes. NT led the analysis and discussed preliminary results with CA, WJ, AH and SN. Participants did not provide feedback on the findings. All authors read and contributed to the manuscript.

## Results

### Study sample characteristics and health insurance coverage

The 10 FGDs at three clinics included 64 participants of whom almost half were experienced (multi-gravid) mothers, with a variety of prior perinatal experiences including giving birth at home, at a migrant clinic or at a government hospital. All of our participants had previously visited or were visiting the migrant clinic to receive ANC and register for birth services. While clinics were on the Thailand side of the border, women came from both sides to receive care. Most of our participants were undocumented migrants who were not eligible for coverage under the Thai government’s Migrant Health Insurance Scheme. Nevertheless women could register to a local non-profit health insurance program known as the M-FUND which was developed with one of the premises to ensure coverage for emergency birth services [7, 18]. Women with M-FUND health insurance were assured that they would have coverage if advanced care at a Thai or Myanmar hospital was required. By registering for ANC, birth services and the M-FUND, women embarked on a pathway for utilizing care for complicated or uncomplicated births.

Pregnant women in our study described availability of maternal health services, accessibility of maternal health service and choice of appropriate care.

### An informal network approach to learning about available maternal health services

When considering availability to maternal health services, some women drew from their past experiences of seeking other types of healthcare from migrant clinics as well as accompanying family members. One woman describes, “I used to follow my mother when I was young. My mother came to this clinic for delivery”.

Participants used their local informal networks to learn about availability and the location of maternal health services from family members, friends and community members including local health workers. Women spoke of following the advice of family members, “I heard it from my aunty that people come to register for their birth here, so that is why I come”. In some cases, they also accompanied a local contact as a woman explains, “One of my neighbors came here and I followed her”. Another woman with a first-time pregnancy followed a community member to the clinic and described how the person had encouraged her to come by asking “Are you not registering for your birth, are you not going?”.

One woman reported that she heard about maternal health services at the migrant clinic from a local health worker, “Before I had a pregnancy. When I came here, I was already 6 months pregnant. I didn’t know there was a clinic here. But the (healthcare worker) told me to come”. Positive reports of birth experiences and perceptions of quality care from other women in the community also shaped women’s desire to seek care at the clinic as, “people talked that the health care worker at (the clinic) takes very good care of the patients”.

### Accessibility: travelling for antenatal and birth services

Women utilized multiple methods of transport to arrive at the clinics including: walking, motorcycle (taxi and personal), car (taxi), boat, bicycle, boat and tractor (taxi). There was a significant range in travel times ranging from 5 min to 4 h. Several women reported walking for up to 2 h to arrive at the clinic. The journeys of women who crossed the border to receive care were often lengthier with the longest being 4 h from home to the clinic, including half an hour at the bridge to apply for a border pass.

Women described how they had travelled to the clinic. For women living on the Thai side of the border, the journey was more straightforward and often required only a single mode of transport or the combination of walking and a taxi ride to arrive at the clinic. One woman described, “I came here by bicycle with my husband… I sat on the back while my husband pedalled”.

Women who lived on the Myanmar side had to make the international border crossing and then continue on to the clinic. The Thailand–Myanmar border is demarcated by the Moei river, a long narrow body of water (Fig. 1). There exists one official border crossing with a large bridge as well as multiple informal crossings where women can come across by boat. Often women used one mode of transport to reach the border, a second to cross it and a third to travel to the clinic. One woman explained that she walked from her home and then “I came across the river by boat. I then came here (to the clinic) by car which took me 15 min”.

When asked about accessibility and getting to care, women did not identify long walks as difficult, even those who walked for 2 h. When probed, participants maintained that it was normal to walk long distances for health services and were very matter of fact about physically navigating this environment to access care. Additionally, participants did not report any work or familial responsibilities that prevented them from coming to the ANC at the clinic.

Travel during the rainy season was sometimes more complex, and compounded challenges caused by the Thailand–Myanmar official bridge crossing. Several women who had previously given birth described the logistical cross-border transportation challenges that they experienced during their labour. One woman registered to give birth at a border clinic in Thailand but ended up going to Myawaddy hospital in Myanmar. She described, “It was the middle of the night and it was difficult to cross (the border) in the rainy season so I had to deliver at Myawaddy hospital”. The woman experienced labour onset in the evening for her first two children and could not cross the border to give birth. The large bridge crossing is closed at night.

Women also described how they would strategically assess where to go to give birth depending on where they were during the onset of labour and the weather conditions, “I think if I began to have labour pain in Myanmar, I would go to the place which is closest to me. We have difficulty with the roads during the rainy season. If not, it’s easy for us to come here with a motorbike”.

Given the challenge of transportation logistics, one woman delivered enroute, “I delivered on the way, in the tractor coming to the clinic. I couldn’t help it”. Other women acknowledged that not arriving at the clinic in time for delivery was a possibility and one expressed, “some people prefer to deliver at home. Some people are used to coming here so they come. For some they gave birth on the way (to the clinic).”

### Choosing appropriate care

For many participants choice of alternative antenatal and birth care, beyond the migrant clinic, was constricted by limited options of care providers who have formal training. When asked about other places they could seek maternal healthcare many indicated that the clinic they were visiting is the only one they knew about. In the voice of some participants, “We have only this one”.

Some women weighed up the option of going to the migrant clinics instead of giving birth at home or in the community and cited higher levels of care in deciding to go to the clinics. One woman explained, “They have a good system of taking care of mother and child, if compared to the place where I live. So, instead of giving birth in the place where I live, I feel more secure to give birth here”. Another noted, “It is not a hundred percent reliable to cut off the umbilical cord at home. It is more reliable here. That’s why I decided to come”. Women expressed that the migrant clinic was better equipped to support them if they had any complications during birth. One participant explained, “I am afraid of some difficulties that would happen if I was there [at home]. But if I am in the hands of a health worker here, I know they have enough medicine. So that, I think is good for me”.

Several women noted that some limited maternal health services were available at the village level. A few women living in Myanmar villages indicated that health workers came to provide vaccinations. In Thailand, some women could also receive ANC from Thai community health centres.

On their respective sides of the border, Thai and Myanmar government hospitals exist which provide maternal healthcare. Depending on where migrant women are living these may be farther than the migrant clinics which are mostly located closely adjacent to the border. Research participants had multiple perspectives on hospital maternal health services. Some knew that additional maternal health services existed within their geographic area, including the local Thai and Myanmar government hospitals, but many had limited knowledge about these care options. For example, when asked about maternal healthcare availability in Myawaddy, the largest town in Myanmar that is close to the Thai border, some women responded, “We heard about it. But we have never been there”. Indeed the distance to this hospital from the clinic of the FGD is some 60 km away.

A few experienced mothers had previously given birth at Thailand and Myanmar government institutions and were thus more knowledgeable about the services. Most had positive birth experiences at government hospitals and a few reported disrespectful care compounded by communication difficulties. Conversations were sometimes rough and care providers appeared impatient amidst language barriers. One woman expressed, “It would be good if we have mutual understanding. We are delivering our babies in a foreign country and automatically feel small or sad.” Overarchingly women perceived that Thai and Myanmar health services were expensive (for those without migrant health insurance) and communication with care providers was sometimes difficult due to language differences and infrequent availability of interpreters. Participants understood that they could access government facilities through a referral from the migrant clinic and their M-FUND insurance but most did not see them as a first choice for accessing ANC and birth care independently.

Cultural appropriateness and affordability of care at migrant clinics helped to make care more accessible for migrant women. At the migrant clinics workers spoke two dialects of Karen language, Sgaw and Pwo as well as Burmese. For migrant patients, being able to get care in a language they understood increased their comfort and influenced their decision to come to the migrant clinic for maternal care. A participant explained, “As for me culturally we speak the same language which is comfortable for me”.

Maternal health care services were provided free of charge at the three migrant clinics we visited. Affordability factored into women’s choice on where to get maternal care and one person expressed, “I get free care here. On the other side (of the border), I had to give money to get care”. Another explained, “We have problems with money. This (service here) makes us happy. It is convenient for me coming here because it costs no money”.

## Discussion

Our study provides insights into perceptions of availability and accessibility based on migrant women’s patient experiences. As described elsewhere, women spoke of multiple factors that influenced their decision to seek maternal care and give birth at the migrant clinics including a desire to seek out safe quality care, affordability, language of care, proximity and perceived limited alternative options (Tschirhart et al. 2020). In this article we expand on the context for their decision making.

Women used an informal network approach to learn about maternal health services from family and community members and similar approaches have been observed among other migrant groups seeking healthcare for other health conditions in the region and beyond [19, 20]. This signals a deep sense of trust between community members which may be based on mutual understanding of the experience of being a migrant in this context and the underlying barriers and limitations which shape healthcare access for these groups. Women also spoke of utilizing known healthcare services and relying on trusted healthcare providers who recommended they come to the clinic. The migrant clinics included in this study also provide other health services, which can lead to well known, trusted and tested pathways to healthcare that women can rely on when seeking ANC. The network approach to learning about health services can provide an opportunity for healthcare providers who can find ways of sharing information about their services with key community members in efforts to better reach migrant populations.

For many of the migrant participants opportunities to have their maternal healthcare needs met were limited by the lack of services available in their geographical area and/or an inability to pay fee-for-service at Thai and Myanmar government hospitals. Choice within this context must be understood as a comparison between options that they perceived to be available to them. Ultimately in this context there exist three different options each with different safety and financial outcomes, giving birth at home, paying for care at a government hospital or attending a migrant clinic where services are provided for free. However, some women were only knowledgeable about the migrant clinic and saw it as a binary choice of going to the clinic or birthing at home [18]. Women overwhelmingly expressed that they perceived migrant health clinics to be the most accessible option for them financially and linguistically. Migrant workers in this border region often receive low wages and have limited additional funds to pay for healthcare. Thai hospital services also employ Burmese interpreters to assist Myanmar women but a need to pay for services for migrants without health insurance may dissuade women from seeking care there [21].

Free access to birth care to all migrants regardless of status has been implemented by some countries while other jurisdictions limit the offer to emergency care including emergency birth services [22]. In related interviews with doctors who provide hospital care in this region of Thailand, clinicians expressed concern that migrant women were delaying coming when in labour and urged individuals to come earlier citing concern about potential adverse outcomes [23]. Doctors indicated that they would provide care during a birth emergency, regardless of a women’s ability to pay [23]. However, if this nuanced application of the hospital policy is not well understood within the migrant community, individuals may avoid care if they do not have the necessary funds. It would also be difficult for migrant women to assess whether their condition is serious enough to warrant treatment without pre-payment. While the reasons that migrant women are arriving late for care are not well understood in this context, it is clear from our study that women do not perceive that they are eligible for low-cost hospital care unless they have migrant health insurance or are referred through the migrant clinic. A future study should examine reasons why women arrive late for emergency obstetrics by collecting data from late arrivals and their families.

Some of the travel distances reported by women were extensive with lengthy travel times of up to 4 h but participants identified the clinic as being close by and explained that it was normal to travel long distances to get care. This conceptualization of what is close and normalization of long travel distances may reflect the limited maternal health services in geographic proximity. While participants did not perceive they have any difficulties, long travel distances such as 2 h by motor bike may contribute to delays arriving at the clinic or hospital in time to give birth. In actuality, one woman gave birth on the way to the clinic and there was an acceptance among the participants that this is a distinct possibility. While patients may see long distance walking to appointments as normal practice, researchers in the region have identified potential challenges to care provision such as concerns about long walking combined with the need to fast for some tests such as that for gestational diabetes screening and monitoring.

Our study identified some considerations that are specific to a border region in South-East Asia. Night time border closures of the main bridges and heavy rain directly influenced accessibility. Women had to be flexible with their birth plan and spoke of deciding where to go to give birth based on the time of day as well as in some cases needing to make an alternate plan if the border was closed or weather was poor or lack of transport made it impossible to travel. This need for an intensive situational analysis reflects a type of conditional or fluctuating accessibility, where time of day, weather and transport need to align to make access to care possible. In this context, migrant women need to invest a considerable about of energy in deliberate situational analysis and planning to be able to adapt and adjust their plan. We anticipate that undocumented migrants in other countries around the world also experience conditional or fluctuating accessibility to birth care, especially in regions where it is necessary to travel due to local unavailability of services. Conditions on accessibility to birth services may be layered and cumulative and should be considered within migrants’ specific context by policy makers and care providers seeking to make services more responsive to patient needs. Leaving accessibility to essential services dependant on weather and time of day, leads to global systematic inequities where undocumented migrant women need to perform intensive situational analysis while women in many countries may not have to consider these challenges.

## Conclusions

While access to a skilled birth attendant is valued internationally, undocumented migrant women around the world face additional challenges due to limited entitlements associated with their legal status. Our study along the Thailand–Myanmar border found that migrant women utilized conversations with their informal network to learn where they could receive care. Their opportunities to reach and obtain services were limited by distance, weather, transport and border closures. Long distance walking to obtain care was normalized by participants and yet may contribute to delays in reaching care while in labour.

Overarchingly, access to free or low-cost antenatal and birth was variable and conditional upon availability of transport, seasonal weather and daylight hours due to international nighttime border closures. Accessibility could shift within hours and was highly contextually dependent. Comparative to Thai citizens living in close proximity to health services, accessibility to care for undocumented migrants in our study was less stable and durable.

Based on publications, primarily from migrant populations in high income countries, one would suspect that there is also an accessibility gap among migrant women in low-and-middle income countries with reduced access and utilization of care [24]. Our research findings confirm conditional or variable access contributing to the literature by sharing undocumented migrant women’s perceptions of care accessibility from a low-and-middle income country, which to date has been underreported. This set of diverse barriers are well known by clinicians working on the ground in this context and these qualitative findings complement spatial and epidemiological studies from the same study setting. For example women lost to follow-up (registered to ANC but with an unknown pregnancy outcome) travelled 50% farther than people who had a normal singleton childbirth, and women who delayed antenatal care until the third trimester also travelled 50% farther compared to people who attended in the first trimester [25]. We anticipate that undocumented migrants in other countries may also experience conditional accessibility to birth care, especially where travel is necessary due to limited local services. By underlining the conditional or fluctuating nature of accessibility to antenatal and birth care for migrants, our study emphasizes how barriers intersect to limit opportunities for care and we encourage researchers to consider how accessibility differs over time and space for migrants with limited financial resources.

Participants described their choices for maternal healthcare as limited and there is value within this region and more globally to increase opportunities for undocumented pregnant women to access maternal healthcare. In working to improve access to care for undocumented migrants in low-and-middle income countries, care providers and policy makers should consider engaging with informal community networks to disseminate information about available low-cost antenatal and birth services. Additionally, as birth may be a rapid process there is a need to address care gaps caused by long distances to clinics and travel barriers such as limited motorized transport. Outreach clinics to provide antenatal services to women in their villages, and increase attachment to care, is a promising option that is being explored in this setting, along with travel subsidies.

## Limitations

A limitation of our study is that we only collected data from women who were already accessing healthcare and thus have not fully explored the perspectives of women who decide or who cannot follow antenatal care and who give birth at home. Additionally, most of our participants are undocumented but a few had documentation (10-year card) to stay in Thailand.

## Data Availability

The datasets generated and/or analyzed during the current study are not publicly available as it was not of the consent and ethical approval but are available from the corresponding author on reasonable request.

## Abbreviations

ANC: Antenatal care
FGD: Focus group discussion
M-FUND: The Migrant Fund
MHIS: The Migrant Health Insurance Scheme
NGO: Non-government organization
SEZ: A special economic zone
SSS: The Social Security Scheme
